# The use of neural networks to determine factors affecting the severity and extent of retinopathy in preterm infants

**DOI:** 10.1186/s40942-025-00650-z

**Published:** 2025-03-14

**Authors:** Mohammad Reza Mazaheri Habibi, Azadeh JafariMoghadam, Narges Norouzkhani, Elham Nazari, Bahareh Imani, Azam Kheirdoust, Seyed Ali Fatemi Aghda

**Affiliations:** 1 Department of Health Information Technology, Varastegan Institute for Medical Sciences, Mashhad, Iran; 2 Department of Health Information Technology and Management, School of Allied Medical Sciences, Shahid Beheshti University of Medical Sciences, Tehran, Iran; 3 Basic Sciences Research Institute, Mashhad University of Medical Sciences, Mashhad, Iran; 4 Department of Pediatrics, Mashhad University of Medical Sciences, Mashhad, Iran; 5 Department of Medical Informatics, Faculty of Medicine, Mashhad University of Medical Sciences, Mashhad, Iran; 6 Fakher Mechatronic Research Center, Kerman University of Medical Sciences, Kerman, Iran; 7 Student Research Committee, School of Health Management and Information Sciences, Iran University of Medical Sciences, Tehran, Iran; 8 Research Center for Health Technology Assessment and Medical Informatics, School of Public Health, Shahid Sadoughi University of Medical Sciences, of Medical Sciences, Yazd, Iran

**Keywords:** Retinopathy, Premature infant, Degree of retinopathy, Disease extent, Artificial neural network

## Abstract

**Background:**

Retinopathy of prematurity (ROP) is a leading cause of visual impairment and blindness in preterm infants. Early identification of key risk factors is essential for effective screening and timely intervention. This study utilizes an artificial neural network (ANN) to analyze and identify the most influential factors affecting the severity and extent of ROP in preterm neonates.

**Methods:**

This descriptive-analytical study was conducted on 367 preterm infants in Bojnord, Iran, in 2021. The study examined multiple variables, including sex, history of multiple births, number of prior abortions, type of pregnancy and delivery, gestational age, oxygen therapy, severity of retinopathy, and disease extent within the retina. Statistical analyses were performed using one-way analysis of variance (ANOVA), Pearson’s correlation coefficient, and an ANN to determine the relationships between independent variables and ROP progression.

**Results:**

The findings indicate that the severity of ROP was significantly associated with the type of pregnancy, gestational age, birth weight, and postnatal age (*P* < 0.05). Similarly, disease extent was significantly correlated with maternal parity, gestational age, birth weight, and postnatal age (*P* < 0.05). Among all factors examined, postnatal and gestational age exhibited the highest coefficient effects on ROP severity and disease extent. Additionally, follow-up evaluations revealed that infant age and birth weight were crucial in disease progression.

**Discussion:**

The results suggest that targeted interventions focusing on gestational age and neonatal weight may significantly reduce the incidence and severity of ROP in preterm infants. Integrating ANNs enhances predictive accuracy, enabling early diagnosis and improved clinical outcomes.

**Conclusion:**

The findings of this study contribute to the advancement of ROP screening and treatment strategies in preterm neonates. Future research should focus on multi-center studies with larger sample sizes to refine predictive models and identify additional risk factors influencing ROP progression.

## Introduction

Retinopathy of prematurity (ROP) is a retinal vascular disease that occurs in preterm infants due to the incomplete development of retinal blood vessels at birth. The severity of ROP varies from mild, self-limiting cases to severe forms, leading to retinal detachment and blindness. The incidence of ROP in Iran has been reported to range from 5.6 to 42%, while in other countries, it varies from 14.4 to 47.2% [1–3]. According to data from the United States, ROP affects approximately 1100 to 1500 neonates annually [2].

In 1984, an international classification system was introduced to categorize ROP based on the extent of the disease (zones), stage of vascular involvement, and severity [4]. Since the late 1980s, significant advancements have been made in ROP management by introducing various treatment modalities. Initially, cryotherapy was widely used, but laser photocoagulation has become the standard treatment [5, 6].

ROP is one of the leading causes of childhood visual impairment and blindness worldwide, particularly in developing countries [7–9]. Although in the past, the lack of systematic screening programs delayed the timely diagnosis and management of ROP, today, standardized screening guidelines have enabled early detection and treatment [10, 11]. Various strategies, including improving neonatal intensive care unit (NICU) management, optimizing oxygen therapy, and implementing systematic screening programs, have been developed to reduce the incidence and complications of ROP.

Identifying key risk factors associated with ROP is essential for its prevention, early detection, and appropriate intervention [12, 13]. Previous studies have examined multiple contributing factors and their correlation with the severity of the disease [9, 14–17]. Three major risk factors consistently associated with ROP include short gestational age, low birth weight, and prolonged exposure to supplemental oxygen [18–22]. Other contributing factors include multiple gestations, the need for respiratory support, and prolonged mechanical ventilation [23].

Given the critical importance of identifying factors influencing ROP incidence and severity, this study will analyze the relationship between these factors and ROP occurrence using artificial neural networks (ANN). Machine learning approaches have shown promise in medical diagnostics, and their application in ROP risk prediction could enhance early screening efforts and improve clinical outcomes.

This descriptive and analytical study was conducted on 367 preterm infants referred to Bent Alhoda Center at Imam Ali Clinic in Bojnord, Iran, in 2021. All participants were suspected of having ROP and were monitored until the completion of retinal vascularization. Infants requiring treatment underwent laser therapy following comprehensive ophthalmologic examinations. Given the critical role of oxygen therapy in the pathogenesis of ROP, the duration of oxygen administration was categorized into three groups based on the neonatologists’ clinical assessment: less than 10 days, between 10 and 30 days, and more than 30 days. Relevant clinical data were extracted from hospital records and systematically recorded for analysis.

The inclusion criteria for this study encompassed preterm birth and a confirmed diagnosis of ROP. Various independent variables were examined, including infant gender, history of multiple births, number of prior abortions, type of pregnancy (spontaneous or assisted), mode of delivery (vaginal or cesarean section), gestational age at birth, neonatal hospitalization status, oxygen therapy duration, type of treatment, and the severity and extent of retinal involvement. The dependent variables included ROP stage and disease extent within the retina.

The classification of the ROP stage follows the international grading system, which ranges from stage 0 (complete vascularization without pathological changes) to stage 5 (total retinal detachment with severe visual impairment). The extent of the disease is determined by the location of vascular involvement, categorized into three anatomical zones. Zone 1, the innermost circular region surrounding the optic nerve and macula, represents the most critical area for vision. Zone 2 extends from Zone 1 toward the peripheral retina, where the most severe cases of ROP typically occur, particularly near the temporal region. Zone 3, the outermost peripheral region, is the least affected area associated with milder disease forms.

Statistical analyses were conducted using SPSS version 25, employing a combination of descriptive and inferential statistical methods, including the Kolmogorov-Smirnov normality test, chi-square test, one-way analysis of variance (ANOVA), Pearson correlation coefficient, and ANNs. Integrating machine learning techniques, particularly neural networks, aimed to enhance the predictive accuracy in identifying high-risk infants and determining significant correlations between clinical variables and ROP development.

## Results

The results of this study are presented in two main sections: descriptive analysis, which outlines the demographic and clinical characteristics of premature infants diagnosed with ROP, and analytical analysis, which examines the relationship between ROP severity, disease extent, and various clinical and demographic variables. Additionally, the study assesses the influence of these variables on the incidence of ROP through statistical modeling.

### Descriptive analysis

The demographic and clinical characteristics of the study population are summarized in Table 1. The findings indicate that most infants were male (54.2%) and singletons (67.3%). A significant proportion of mothers (64.6%) had no history of prior abortion, and in 70.6% of cases, pregnancy occurred naturally without assisted reproductive techniques. Regarding mode of delivery, cesarean section was the predominant method (73%). Regarding parity, 60.5% of mothers had between one and two previous children.

**Table 1:** Descriptive characteristics of study variables (*n* = 367)

Variables	Variables Subset	Number	Percentage	Mean And SD
Gender	Girl	164	44.7	
	Boy	199	54.2	
	No Response	4	1.1	
History	Singleton	247	67.3	
	twin	104	28.3	
	Triplet	4	1.1	
	No Response	12	3.3	
The Number of Maternal Abortions	0	237	64.6	
	1	62	16.9	
	2	15	4.1	
	More than 2	53	14.4	
Type of Pregnancy	Normal	259	70.6	
	Infertility treatment	34	9.3	
	Medication Treatment	3	0.8	
	No Response	71	19.3	
Type of Parturition	Natural	84	22.9	
	Cesarean Section	268	73	
	No Response	15	4.1	
The Number of Mothers’ Pregnancies	Children1-2	222	60.5	
	Children3-4	93	25.3	
	More than 4 children	16	4.4	
	No Response	36	9.8	
Length of Pregnancy of Mothers by Week	25–30	44	12	
	30–35	242	65.9	
	35–40	77	21	
	No Response	4	1.1	
The Condition of The Newborn	Yes	264	71.9	
	No	103	28.1	
Oxygen	Yes	269	73.3	
	No	11	3	
	No Response	87	23.7	
Days of Receiving Oxygen	Less than 10 days	263	71.7	
	Between 10–30 days	6	1.6	
	No Response	98	26.7	
Type of Treatment	IVB^1^	2	0.5	
	F/U^2^	302	82.3	
	No Response	63	17.2	
Mother’s Age				29.19 ± 6.68
Infant’s Age by Weeks				3.45 ± 37.71
Baby’s Birth Weight in Grams				1894.21 ± 565.64
Number of Hospitalization Days				9.44 ± 7.57
The Extent of the Disease	0	63	17.2	
	Zone1	9	2.4	
	Zone2	81	22.1	
	Zone3	214	58.3	
Degree of Retinopathy	0	286	77.9	
	1	49	13.4	
	2	32	8.7	

**IVB 1**: Intra vitreous injection. The treatment method is for patients who have entered the advanced phase and needed intervention.

**F/U2**: Follow-up for patients who entered the advanced phase but did not need intervention. In such a way that the duration of visits was made closer so that intravitreous injection can be used in case of disease progression

Gestational age at birth varied among the study participants, with 65.9% of infants born between 30 and 35 weeks of gestation. Oxygen therapy, a key risk factor for ROP, was administered to 71.7% of neonates for less than 10 days. The most common treatment approach (82.3%) was management through follow-up visits, indicating that most cases did not require immediate intervention.

The mean maternal age was approximately 29 years, while the mean birth weight of the infants was around 1800 g. The average gestational age at birth was reported as 37 weeks, and the mean duration of neonatal hospitalization was 9 days.

Regarding ROP severity and extent, Zone 3 was the most commonly affected retinal region, accounting for 58.3% of cases (214 infants). Furthermore, the highest proportion of infants (77.9%, *n* = 286) were classified as Stage 0, indicating complete retinal vascularization without pathological changes.

### Analytical analysis

The relationship between ROP severity, disease extent, and independent clinical variables is detailed in Table 2. This section explores the statistical associations between ROP progression and key perinatal factors, including gestational age, birth weight, oxygen therapy duration, and neonatal hospitalization parameters. Additionally, the impact of these variables on ROP incidence was quantified through regression models and ANN analysis.

**Table 2:** Investigating the relationship between degree of retinopathy and extent of the disease with other independent variables

Variable Name		Gender	History	Number of Abortions	Type of Pregnancy	Type of Parturition	Number of Pregnancies	Length of Pregnancy	Mother’s Age	Baby’s Weight	Baby’s Age	Received Oxygen Status	The Number of Days Receiving Oxygen
Degree of retinopathy		Chi-square	Chi-square	Chi-square	Chi-square	Chi-square	Anova	Anova	Anova	Anova	Anova	Chi-square	Chi-square
		Value:7/720	Value:6/612	Value:5.909	Value:17/089	Value:0/368	Df:3	Df:3	Df:3	Df:3	Df:3	Value:5.777	Value:1.145
		Df:3	Df:6	Df:12	Df:6	Df:3	p-value:0.284	p-value: <0.001	p-value:0.847	p-value: <0.001	p-value: <0.001	Df:3	Df:3
		p-value:0.052	p-value:0.358	p-value:0.921	p-value:0.009	p-value:0.947	F = 1.272	F = 30.025	F = 0.269	F = 15.955	F = 16.892	p-value:0.123	p-value:0.766
**Extent of the Disease**		**Chi-square**	**Chi-square**	**Chi-square**	**Chi-square**	**Chi-square**	**Anova**	**Anova**	**Anova**	**Anova**	**Anova**	**Chi-square**	**Chi-square**
		Value:0/357	Value:2/493	Value:12.033	Value:5/174	Value:0/292	Df:2	Df:2	Df: 2	Df:2	Df:2	Value:2.057	Value:2.891
		Df:2	Df:4	Df:8	Df:4	Df:2	p-value: 0.021	p-value: <0.001	p-value:0.306	p-value: <0.001	p-value: <0.001	Df:2	Df:2
		p-value:0.837	p-value:0.646	p-value:0.15	p-value:0.270	p-value:0.864	F = 3.885	F = 80.323	F = 1.187	F = 59.732	F = 87.453	p-value:0.357	p-value:0.236

### Analytical section

The analytical phase of this study aimed to investigate the relationships between ROP severity, disease extent, and various independent clinical and demographic variables. The statistical analyses were conducted following an assessment of data normality, which determined the use of parametric tests for further examination. As shown in Table 2, the findings indicate that the type of pregnancy, gestational age, birth weight, and postnatal age of the infant were significantly correlated with ROP severity. Similarly, maternal parity, gestational age, birth weight, and infant age were significantly associated with the extent of retinal involvement. Notably, gestational age, birth weight, and postnatal age emerged as key factors influencing both ROP severity and disease extent, highlighting their crucial role in the pathogenesis and progression of the disease.

To further analyze the impact of these factors on ROP severity and disease extent, a Multilayer Perceptron (MLP) neural network model was employed. The model was structured with two hidden layers, a learning rate of 0.001, ten iterations, and a training-to-testing ratio of 70:30. The results of the ANN analysis, presented in Table 3, provide insights into the relative importance of each variable in predicting ROP severity and disease extent. The findings demonstrate that postnatal and gestational age exhibited the highest coefficient effects, reinforcing their significance in disease progression.

**Table 3:** Coefficient effect of variables on the extent of the disease and degree of retinopathy

Variables	Normalized importance extent of the disease	Importance extent of the disease	Normalized importance degree of retinopathy	Importance degree of retinopathy
History	0.036	13.60%	0.044	12.90%
Pregnancy	0.039	14.50%	0.029	8.60%
Type of Parturition	0.014	5.10%	0.012	3.40%
Received Oxygen Status	0.027	10.20%	0.019	5.70%
Days of Receiving Oxygen	0.024	9.00%	0.016	4.80%
Gender	0.029	10.80%	0.031	9.00%
Number of Abortions	0.083	31.00%	0.059	17.30%
Mother’s Age	0.076	28.40%	0.087	25.60%
Gestational Age in Weeks	0.186	69.70%	0.224	65.90%
Baby’s Birth Weight in Grams	0.142	53.30%	0.07	20.60%
Baby’s Age at First Visit in Weeks	0.267	100.00%	0.34	100.00%
Number of pregnancies	0.078	29.30%	0.07	20.80%

The predictive performance of the MLP neural network was evaluated, yielding an accuracy rate of 79.2%, suggesting a robust predictive capability. The effectiveness of ROP management strategies over multiple follow-up visits was assessed through longitudinal analysis. The frequency distribution of ROP severity and disease extent across follow-up visits is detailed in Table 4. The findings reveal a notable reduction in disease severity and extent between the second and fourth follow-up visits, indicating the effectiveness of timely interventions in disease stabilization and regression.

**Table 4:** Frequency of degree of retinopathy and extent of disease in different follow-ups

Follow-up 1	Degree of retinopathy	0	286	77.9
		1	49	13.4
		2,3	32	8.7
	Extent of the disease	Zone1	72	19.6
		Zone2	81	22.1
		Zone3	214	58.3
Follow-up 2	Degree of retinopathy	0	301	82
		1	48	13.1
		2,3	18	4.9
	Extent of the disease	Zone1	184	50.1
		Zone2	32	8.7
		Zone3	151	41.2
Follow-up 3	Degree of retinopathy	0	351	95.6
		1	12	3.3
		2,3	4	1.1
	Extent of the disease	Zone1	326	88.8
		Zone2	7	1.9
		Zone3	34	9.3
Follow-up 4	Degree of retinopathy	0	364	99.2
		1	1	0.3
		2,3	2	0.5
	Extent of the disease	Zone1	364	99.2
		Zone2	1	0.3
		Zone3	2	0.5

Further analysis in Table 5 explores the relationship between infant age and birth weight with ROP severity and disease extent across follow-up visits. The results suggest a consistent association between higher birth weight and lower ROP severity and an inverse relationship between increasing infant age and disease extent.

**Table 5:** Investigating the relationship between the age and weight of the infant with the degree of retinopathy and the extent of the disease in different follow-ups

		Age	Weight
Follow-up 1	Degree of retinopathy	**Anova**	**Anova**
		Df: 3	Df: 3
		P-value: <0.001	P-value: <0.001
		F = 16.892	F = 15.955
	Extent of the disease	**Anova**	**Anova**
		Df: 2	Df: 2
		P-value: <0.001	P-value: <0.001
		-	F = 59.732
Follow-up 2	Degree of retinopathy	**Anova**	**Anova**
		Df: 3	Df: 3
		P-value: 0.406	P-value: 0.003
		F = 0.974	F = 4.979
	Extent of the disease	**Anova**	**Anova**
		Df: 2	Df: 2
		P-value: 0.004	P-value: <0.001
		F = 5.799	F = 8.893
Follow-up 3	Degree of retinopathy	**Anova**	**Anova**
		Df: 51	Df: 3
		P-value: 0.63	P-value: 0.155
		F = 46.855	F = 1.874
	Extent of the disease	**Anova**	**Anova**
		Df: 2	Df: 1
		P-value: 0.48	P-value: 0.003
		F = 3.318	F = 10.336

Pearson’s correlation coefficient was calculated for the second and third follow-up visits to quantify the relationships between infant age, birth weight, ROP severity, and disease extent. The analysis revealed a significant negative correlation between birth weight and ROP severity in both the second follow-up (*P* < 0.001, *r*=-0.293) and third follow-up (*P* = 0.021, *r*=-0.395), indicating that higher birth weight was associated with reduced ROP severity.

Similarly, a significant correlation was observed between infant age and birth weight with disease extent, with statistical significance in both the second follow-up (birth weight: *P* < 0.001, *r* = 0.315; age: *P* = 0.002, *r* = 0.234) and the third follow-up (birth weight: *P* = 0.003, *r* = 0.500; age: *P* = 0.020, *r* = 0.372). These results suggest that as infants grow older and gain weight, the extent of retinal disease involvement diminishes, further emphasizing the protective role of gestational maturity and postnatal growth in ROP progression.

## Discussion

ROP remains a significant clinical concern due to its potential complications, particularly vision loss in preterm infants. Early identification of risk factors associated with ROP severity and disease extent is essential for timely intervention and preventing adverse visual outcomes. In the present study, the correlation between key clinical variables—such as infant age, gender, and perinatal factors—with ROP severity and disease extent was systematically analyzed. Moreover, the relative importance of these factors was assessed using statistical modeling to determine their coefficient effects.

The relationship between ROP severity and disease extent was evaluated concerning 12 independent variables, including gender, pregnancy history, number of abortions, type of pregnancy, mode of delivery, number of pregnancies, gestational age, maternal age, birth weight, postnatal age, oxygen therapy status, and duration of oxygen therapy. The findings revealed that ROP severity was significantly associated with the type of pregnancy, gestational age, birth weight, and postnatal age (*P* < 0.05). Similarly, the extent of disease showed a significant correlation with the number of pregnancies, gestational age, birth weight, and postnatal age (*P* < 0.05).

Further analysis demonstrated that gestational and postnatal age exhibited the highest coefficient effects on ROP severity and disease extent, underscoring their pivotal role in disease progression. Additionally, the effectiveness of treatment strategies was assessed across four follow-up visits, where a notable reduction in ROP severity and disease extent was observed, highlighting the efficacy of timely intervention and disease management.

A study by Shah et al. in Singapore retrospectively analyzed ROP’s frequency and risk factors in infants with very low birth weight (VLBW) over 14 years (1988–2001). Using the international classification of ROP, they investigated premature infants and identified significant risk factors through logistic regression analysis, including maternal preeclampsia, low birth weight, pulmonary hemorrhage, duration of mechanical ventilation, and continuous positive airway pressure (CPAP) therapy [19]. Their findings align with the present study, reinforcing the critical role of birth weight and respiratory support in ROP pathogenesis.

The application of artificial intelligence (AI) in ROP screening and risk assessment has gained increasing attention in recent years. In a study by Aaron S. Coyner et al., conducted between February 1, 2019, and June 30, 2021, an AI-driven vascular severity model was developed to predict treatment-requiring ROP (TR-ROP). Retinal fundus images from Indian neonates undergoing routine medical screening were analyzed remotely, with an AI-derived vascular severity score (VSS) extracted from images obtained after 30 weeks of postmenstrual age. Using 5-fold cross-validation, logistic regression models incorporating gestational age and VSS were trained to predict TR-ROP, demonstrating the potential utility of AI-assisted diagnostics in remote screening programs [20].

Several studies have examined the clinical and demographic risk factors associated with ROP across different populations. Naderian et al. investigated ROP incidence and its relationship with gestational age, birth weight, and oxygen therapy duration in neonates admitted to Shahid Beheshti and Al-Zahra medical centers in Isfahan. Their findings indicated that infants receiving oxygen therapy for more than 30 days had a higher incidence of ROP than those treated for only 10 days. However, while ROP occurrence was significantly associated with gestational age and birth weight, the duration of oxygen therapy did not have a statistically significant impact on disease incidence [24].

Similarly, Abrishami et al. conducted a study in Mashhad to investigate the relationship between ROP and neonatal hypoxia, hyperoxia, blood oxygen saturation (SpO₂), gestational age, and birth weight. Their findings indicated a relatively high incidence of ROP in this region, with significant correlations between ROP severity, gestational age, birth weight, and Apgar scores [21].

In a related study, Ebrahimi et al. examined 173 premature neonates in Babol City to determine ROP’s incidence and risk factors. Their results highlighted low gestational age, low birth weight, phototherapy, and blood transfusion as significant risk factors for ROP development, while additional associations were observed with supplemental oxygen therapy, acute respiratory distress syndrome, and sepsis [25].

The findings from these studies align with the present study’s results, reinforcing the critical role of gestational age and birth weight as primary determinants of ROP severity and disease extent. Moreover, the emerging role of AI-based predictive models offers promising avenues for enhanced screening, early diagnosis, and targeted interventions in high-risk neonates.

Several studies have explored the risk factors associated with the development and severity of ROP, further reinforcing the present study’s findings. In a study conducted by Yau GS et al. in Hong Kong, 513 newborns were screened between January 2007 and December 2012 using univariate and multivariate logistic regression analyses. The incidence of ROP and type 1 ROP was reported as 18.5% and 3.7%, respectively. Univariate analysis identified several significant risk factors, including lower gestational age, low birth weight, blood transfusion, patent ductus arteriosus, use of non-steroidal anti-inflammatory drugs (NSAIDs), postpartum hypotension, inotrope use, low Apgar scores, sepsis, mechanical ventilation, oxygen supplementation, respiratory distress syndrome, anemia, use of surfactant, and bronchopulmonary dysplasia (*P* < 0.05). In contrast, multivariate logistic regression analysis confirmed that low birth weight, low gestational age, and intraventricular hemorrhage were significant independent risk factors for ROP, while preeclampsia and eclampsia appeared to have a protective effect against ROP development (*P* = 0.02) [22, 26, 27].

Similarly, in a study by Chattopadhyay et al., 50 preterm infants in a teaching hospital in northeastern India were screened for ROP. The incidence of ROP was 44% (22 out of 50 infants), and a strong correlation was found between low birth weight and gestational age at the time of birth with ROP development. After controlling for potential confounding factors, stepwise regression analysis identified apnea as a significant risk factor for ROP progression [28].

In another study conducted by Ahuja et al. in India (2018), 325 preterm infants were screened for ROP, with 106 infants (32.6%) diagnosed with the disease. The frequency of stage 3 ROP was reported as 13.2%. This study’s only statistically significant variable was low birth weight, with an average weight of 1287 g in infants with ROP compared to 1472 g in infants without ROP [29].

A comparative analysis of the present study with prior investigations highlights infant age and birth weight as two consistently emphasized risk factors for ROP development. Additionally, the present study identified significant associations between the type of pregnancy and gestational age with ROP severity, maternal parity and gestational length with disease extent. Notably, the application of ANN modeling in this study further confirmed that infant age and gestational length exhibited the highest coefficient effects on ROP severity and disease extent, reinforcing their pivotal role in disease pathogenesis and progression.

While oxygen therapy duration has been widely reported as a major risk factor for ROP in previous studies, the present study found no significant relationship between prolonged oxygen exposure and ROP incidence. This lack of significance may be attributed to improved neonatal respiratory support, including advancements in mechanical ventilation techniques, stricter oxygen therapy protocols, and enhanced neonatal care strategies that ensure more precise oxygen delivery to preterm infants. Consequently, the traditionally reported impact of prolonged oxygen exposure on ROP development appears to have diminished, underscoring the evolving nature of neonatal care in ROP prevention.

Furthermore, the present study found no significant association between ROP incidence and infant gender or the duration of neonatal hospitalization (*P* > 0.05), suggesting that these variables may not play a direct role in disease progression.

Integrating AI in ROP screening and diagnosis has demonstrated significant potential in improving cost-effectiveness, accessibility, and accuracy compared to traditional telemedicine and ophthalmology-based approaches [30]. In a study by Qiaowei Wu et al. involving data from 815 infants, deep learning models exhibited high accuracy and strong generalizability in predicting ROP, highlighting the growing role of AI-driven methodologies in neonatal ophthalmic care [31].

A key strength of the present study was utilising an MLP neural network to comprehensively assess the impact of multiple clinical and demographic factors on ROP severity and disease extent. Using machine learning algorithms provided valuable insights into the relative importance of various risk factors, reinforcing the predictive value of gestational age and postnatal age in disease progression. Given the promising results, future research should explore additional machine learning techniques, incorporating a broader range of variables to enhance predictive accuracy and clinical applicability.

Despite these strengths, the study had certain limitations. The primary constraint was using data from a single medical center, which may limit the generalizability of the findings. Expanding the dataset to include multiple centers with diverse neonatal populations would provide a more comprehensive and representative analysis of ROP risk factors. Additionally, future studies should investigate the impact of different oxygen delivery methods (invasive vs. non-invasive) on ROP progression and treatment outcomes, as this could provide further insights into optimizing respiratory management strategies in preterm infants.

## Conclusion

The findings of this study highlight the significant role of gestational age and postnatal age in determining the severity and progression of ROP in preterm infants. This study underscores the importance of early screening and individualized treatment strategies for high-risk neonates by identifying key risk factors, including birth weight and gestational age. The results suggest that timely interventions tailored to an infant’s risk profile could improve disease management and outcomes.

Further research utilizing larger, multi-center datasets are warranted to validate these findings and explore additional contributing factors. Expanding the scope of studies may enhance the precision of ROP screening protocols and optimize treatment approaches, ultimately improving visual prognosis and long-term outcomes for preterm infants.

## Data Availability

The dataset and statistical analysis plan are available upon reasonable request from the corresponding author.

## Abbreviations

ROP: Retinopathy of Prematurity
GA: Gestational Age
BW: Birth Weight
AI: Artificial Intelligence
MLP: Multilayer Perceptron
SPSS: Statistical Package for the Social Sciences
TR-ROP: Type 1 Retinopathy of Prematurity
ANN: Artificial Neural Network
NICU: Neonatal Intensive Care Unit
VLBW: Very Low Birth Weight
CPAP: Continuous Positive Airway Pressure
VSS: Vascular Severity Score
NSAIDs: non-steroidal anti-inflammatory drugs
